# A Pro-Tumorigenic Effect of Heparanase 2 (Hpa2) in Thyroid Carcinoma Involves Its Localization to the Nuclear Membrane

**DOI:** 10.3389/fonc.2021.645524

**Published:** 2021-04-20

**Authors:** Itai Margulis, Inna Naroditsky, Miriam Gross-Cohen, Neta Ilan, Israel Vlodavsky, Ilana Doweck

**Affiliations:** ^1^ Department of Otolaryngology, Head and Neck Surgery, Carmel Medical Center, Haifa, Israel; ^2^ Department of Pathology, Rambam Health Care Campus, Haifa, Israel; ^3^ Technion Integrated Cancer Center, Rappaport Faculty of Medicine, Technion, Haifa, Israel; ^4^ Rappaport Faculty of Medicine, Technion, Haifa, Israel

**Keywords:** thyroid carcinoma, heparanase 2, immunostaining, localization, nuclear membrane, metastasis

## Abstract

Activity of the endo-beta-glucuronidase heparanase, capable of cleaving heparan sulfate (HS), is most often elevated in many types of tumors, associating with increased tumor metastasis and decreased patients’ survival. Heparanase is therefore considered to be a valid drug target, and heparanase inhibitors are being evaluated clinically in cancer patients. Heparanase 2 (Hpa2) is a close homolog of heparanase that gained very little attention, likely because it lacks HS-degrading activity typical of heparanase. The role of Hpa2 in cancer was not examined in detail. In head and neck cancer, high levels of Hpa2 are associated with decreased tumor cell dissemination to regional lymph nodes and prolonged patients’ survival, suggesting that Hpa2 functions to attenuate tumor growth. Here, we examined the role of Hpa2 in normal thyroid tissue and in benign thyroid tumor, non-metastatic, and metastatic papillary thyroid carcinoma (PTC) utilizing immunostaining in correlation with clinicopathological parameters. Interestingly, we found that Hpa2 staining intensity does not significantly change in the transition from normal thyroid gland to benign, non-metastatic, or metastatic thyroid carcinoma. Remarkably, we observed that in some biopsies, Hpa2 is accumulating on the membrane (envelop) of the nucleus and termed this cellular localization NM (nuclear membrane). Notably, NM localization of Hpa2 occurred primarily in metastatic PTC and was associated with an increased number of positive (metastatic) lymph nodes collected at surgery. These results describe for the first time unrecognized localization of Hpa2 to the nuclear membrane, implying that in PTC, Hpa2 functions to promote tumor metastasis.

## Introduction

Heparanase is an endo-beta-glucuronidase capable of cleaving heparan sulfate (HS) side chains of heparan sulfate proteoglycans (HSPGs). HSPGs are highly abundant in the extracellular matrix (ECM) and assist in assembling the major protein constituents of the ECM (i.e., laminin, fibronectin, collagen-IV, etc.) into a three-dimensional, non-soluble, thick matrix that provides structural support and biochemical cues to various cell types. Cleavage of HS by heparanase thus results in remodeling of the ECM. These structural and biochemical alterations are expected to exert a profound impact on cell behavior including, among others, cell differentiation, proliferation, migration and invasion. The latter is most often associated with increased metastatic capacity of tumor cells and augmented entry of inflammatory cells (i.e., T-cells, macrophages, NK-cells) to sites of inflammation (1–3). Heparanase also cleaves HSPGs on the cell surface (i.e., syndecans), affecting their ability to function as co-receptors in signaling pathways (4). This, and other mechanisms utilized by heparanase to promote tumorigenesis (5–9), have turned this enzyme into a promising drug target and heparanase inhibitors are currently being evaluated in clinical trials as anti-cancer drugs (10, 11). Elevated levels of heparanase were documented in an increasing number of human carcinomas and hematological malignancies, often associating with increased tumor metastasis and shorter survival rates (5–9). In head and neck (H&N) cancer, heparanase expression is inversely correlated with patient status (12). Moreover, the cellular localization of heparanase had a profound impact on the patients’ outcome. Thus, cytoplasmic staining of heparanase inversely correlated with patient survival and predicted poor prognosis, whereas nuclear heparanase predicted a favorable outcome (12).

Heparanase 2 (Hpa2) is a close homolog of heparanase that lacks intrinsic HS-degrading activity, the hallmark of heparanase, yet retains the capacity to bind HS with high affinity (13). The consequences of HS binding and clustering by Hpa2 are not entirely clear but may lead to inhibition of heparanase activity and uptake (13). Moreover, studies revealed a physical association between Hpa2 and heparanase proteins (13), providing an additional route by which Hpa2 can inhibit heparanase enzymatic activity. The role of Hpa2 in cancer is largely unknown. In H&N cancer, high levels of Hpa2 were associated with prolonged patients’ survival and decreased tumor cell dissemination to regional lymph nodes (13, 14). Notably, overexpression of Hpa2 in H&N cancer cells resulted in a marked decrease in tumor growth, associating with a prominent reduction in tumor vascularity (15), and further supporting the notion that Hpa2 functions to attenuate H&N cancer.

Thyroid cancer is the most common endocrine malignancy with increasing incidence. Papillary thyroid carcinoma (PTC) is the most common pathology of thyroid cancer. It has a high propensity to lymph node (LN) metastases, and gross metastases may be seen in up to 35% of patients at the time of diagnosis (16, 17). Surgical resection is the mainstay of treatment, and worldwide guidelines specify the extent of surgery needed (17, 18). Some clinical and pathological risk parameters have been reported as important predictors of disease recurrence and patient survival, and for decision-making strategies in treating metastatic PTC. These parameters include patients’ age, tumor (T) stage, extrathyroidal extension (ETE), positive surgical margins, extracapsular extension, lymphovascular invasion, total nodal yield, and the number of positive nodes (16–19).

Given that Hpa2 exhibits different expression patterns in various tissues and their respective carcinomas (20, 21), we examined the expression of Hpa2 in normal thyroid tissue and in benign, non-metastatic, and metastatic PTC in correlation with clinicopathological parameters.

## Materials and Methods

### Study Design

This is a retrospective study of patients diagnosed with PTC and who underwent thyroid surgery, with or without neck dissection, in the Department of Otolaryngology, Head and Neck Surgery at Carmel Medical Center during 2010-2019. Medical records were collected and, according to pathological diagnosis, patients were enrolled in the following study groups:

**Group A**- Patients with benign lesions of the thyroid gland: Goiter and follicular adenoma. These patients underwent hemithyroidectomy or total thyroidectomy.

**Group B-** Patients diagnosed with PTC, localized to the thyroid gland, with no lymph node metastasis. These patients underwent thyroidectomy (hemithyroidectomy or total thyroidectomy). Patients with follicular carcinoma of the thyroid were not included in the current study.

**Group C-** Patients diagnosed with regional metastatic PTC who underwent thyroidectomy and neck dissection (ND) due to lymph node metastasis.

Demographic, imaging, pathology, staging, surgery, and outcome data were collected from medical files. We evaluated the following clinicopathological parameters: patient age, status of the disease, tumor (T), nodal (N) and distant metastasis (M) stage, extra thyroid extension (ETE), perineural and vascular invasion, total number of positive nodes, locoregional and distant recurrence of the disease. The respective paraffin-embedded pathological specimens were withdrawn from the archives and prepared for immunohistochemical analysis.

### Hpa2 Immunostaining

Immunostaining of formalin-fixed, paraffin-embedded 5-micron sections was performed essentially as described (13), utilizing anti-Hpa2 polyclonal antibody #58. Antibody #58 was raised in rabbits against a peptide (^43^DRRPLPVDRAAGLKEKT^59^) mapped at the N-terminus of Hpa2. This peptide was preferred because it exhibits minimal sequence homology with heparanase (22) and the respective antibody was expected and later shown to recognize the wild type (Hpa2c) and alternatively spliced Hpa2 (Hpa2a, Hpa2b) by immunoblotting of cell extracts (22). Moreover, the antibody was found suitable for immunostaining of paraffin sections, and its immunoreactivity was abolished by the peptide, thus granting specificity of the staining (13). Briefly, slides were deparaffinized, rehydrated and endogenous peroxidase activity was quenched (30 min) by 3% hydrogen peroxide in methanol. Slides were then subjected to antigen retrieval by boiling (20 min) in 10 mM citrate buffer, pH 6. Following washes with phosphate-buffered saline (PBS), slides were incubated with 10% normal goat serum (NGS) in PBS for 60 min to block nonspecific binding and incubated (20 h, 4°C) with anti-Hpa2 antibody #58 (13) diluted in blocking solution. Slides were extensively washed with PBS and incubated with a secondary reagent (Envision kit) according to the manufacturer’s (Dako, Glostrup, Denmark) instructions. Following additional washes, color was developed with the AEC reagent (Dako, Glostrup, Denmark), sections were counterstained with hematoxylin and mounted. Immunostained specimens were examined by a senior pathologist and were scored according to the intensity of staining (0-none;1-weak; 2-strong) and the cellular localization of Hpa2 staining (C-cytoplasmic, N-nuclear, NM-nuclear membrane). Images were acquired by Nikon ECLIPSE microscope and Digital Sight Camera (Nikon) with objectives x40, x100.

### Statistical Analysis

All the parameters were analyzed for normal distribution. Correlations between variables were done using the Pearson`s and Spearman`s coefficients of correlation, for parametric and non-parametric groups, respectively. Univariate analyses of disease control for the measured variables were performed by constructing Kaplan Meier curves, and statistical significance between subgroups was tested using the Log-Rank test. For all analyses, p <0.05 is considered significant.

## Results

### Study Design

162 patients were enrolled in the study and were divided into the following study groups (**Table 1**):

**Table 1:** Demographic, pathology, staining intensity and cellular localization of Hpa2 in benign lesions (group A), non-metastatic (group B), and metastatic (group C) thyroid carcinoma.

		Normal thyroid tissue adjacent PTC (n = 44)	Benign tumors (Group A; n = 56)	Non-metastatic PTC (Group B; n = 44)	Metastatic PTC (Group C; n = 62)
		Number of patients	Number of patients (%)	Number of patients (%)	Number of patients (%)
**Age**		53.2 ±16.4	47.95 ± 15.7	53.2±16.4	49.1±18.5
**Gender**	Male	16	23	16	28
	Female	28	33	28	35
**T-Stage**	T1a			17 (39)	11 (18)
	T1b			11 (25)	24 (39)
	T2-4			16 (36)	27 (43)
**N-Stage**	N0			44 (100)	3 (5)
	N1a			0	13 (21)
	N1b			0	46 (74)
**Hpa2** **intensity**	0	10 (22)	8 (14)	9 (21)	10 (16)
	1	13 (30)	27 (48)	19 (43)	33 (53)
	2+3	21 (48)	21 (38)	16 (36)	19 (31)
**Hpa2 cellular** **localization**	0	10 (22)	8 (14)	9 (20)	10 (16)
	c	13 (30)	5 (9)	18 (41)	21 (34)
	c+N	21 (48)	43 (77)	14 (32)	18 (29)
	c+NM	0	0	3 (7)	**13 (21)**

c, cytoplasm; c+N, cytoplasm and nucleus; c+NM, cytoplasm and nuclear membrane. Numbers in bold direct readers to the most important values.

**Group A-** Benign lesions of the thyroid gland. This group included 56 patients (33 females and 23 males) with pathological diagnoses of benign lesions of goiter (42 patients) or follicular adenoma (14 patients). Mean age of patients with goiter was 45.8 (11-70.7), 27 females and 15 males. Fourteen patients with follicular adenoma had a mean age of 54.6 (27.9-73.3), 6 females and 8 males. Forty-two patients (75%) underwent hemithyroidectomy and fourteen patients (25%) underwent total thyroidectomy. Types of tumors were non-toxic nodular and multinodular goiter (37 patients, 66%), toxic goiter/thyrotoxicosis (4 patients, 7%), follicular adenoma (14 patients, 25%), or metaplasia (1 patient, 2%).

**Group B-** Non-metastatic papillary thyroid carcinoma. This group included 44 patients (28 females and 16 males) with pathological diagnoses of PTC without lymph node or distant metastasis. Patient’s age was 53.2 ± 16.4 years (range 21.5-80.4). Twenty-one patients (48%) underwent hemithyroidectomy and twenty-three patients (52%) underwent total thyroidectomy. The follow-up time of group B was 53.7 ± 27 months (range 9.1-107.3).

At the end of follow-up, all patients (44) were alive and with no evidence of disease (NED). Hpa2 staining was also examined in normal thyroid tissues adjacent to the tumor lesions (control tissue).

**Group C-** Metastatic papillary thyroid carcinoma. This group included 62 patients (35 females and 27 males) with a pathological diagnosis of PTC with lymph node metastasis. Patients age was 49.1 ± 18.5 years (range 15.3-86.5). All patients underwent total or complete thyroidectomy. Fifty-eight patients (94%) underwent neck dissection of the central compartment, with or without lateral compartment. The follow-up time of group C was 55.2 ± 27.2 months (range 14-117.2). At the end of the follow-up, 61 patients were alive and 1 patient died of the disease. Fifty-six patients (90%) had no evidence of disease and 5 patients (8%) were alive with disease.

### Immunostaining of Hpa2

**Figure 1:**
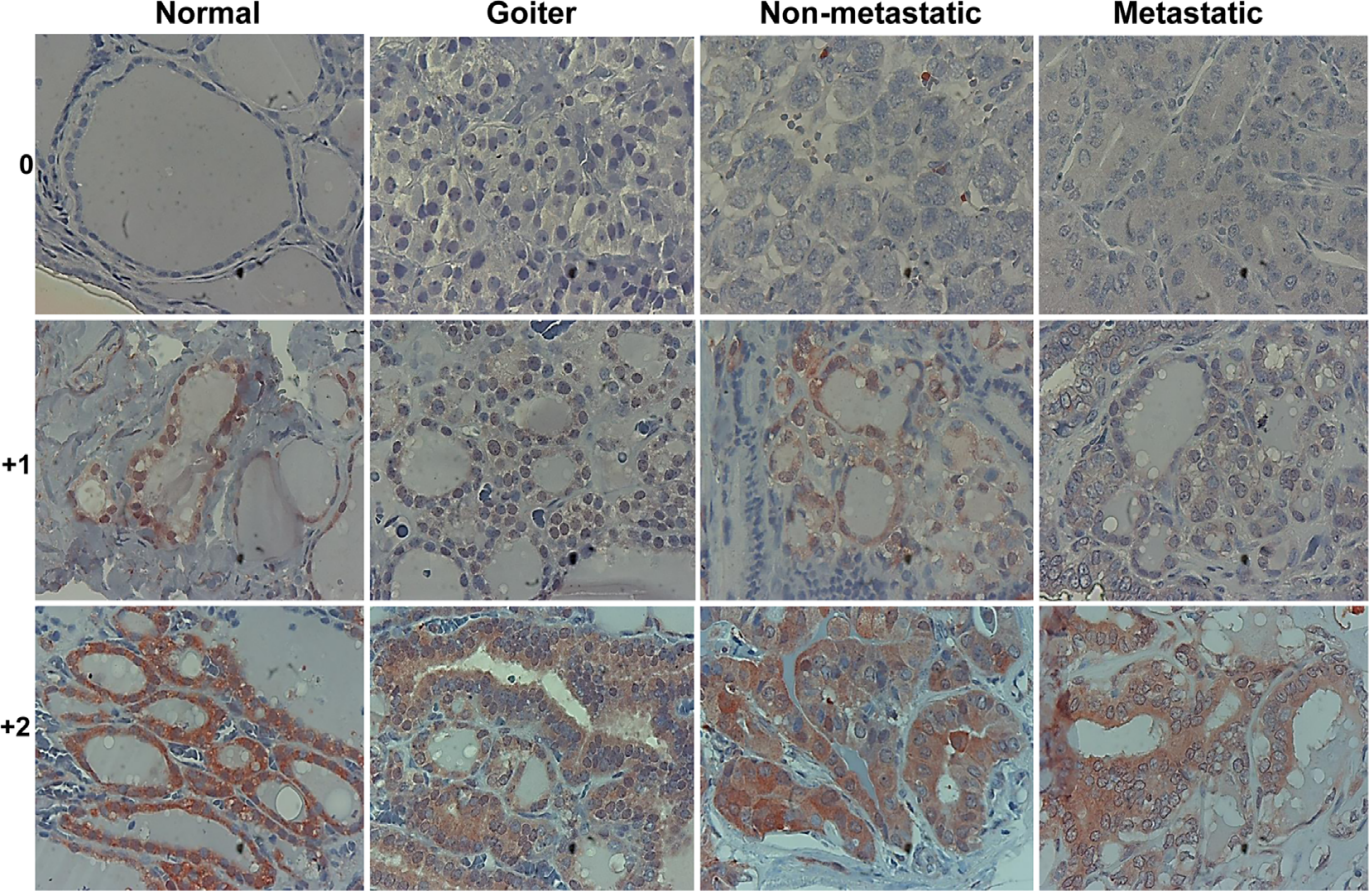
Immunostaining. Five-micron sections of normal thyroid gland adjacent to thyroid carcinoma (left panels), benign (Goiter; second left), non-metastatic (second right), and metastatic thyroid carcinoma (right panels) were subjected to immunostaining applying anti-Hpa2 antibody (#58). Shown are representative photomicrographs of Hpa2-negative (upper panels) and positive biopsies exhibiting weak (+1; middle panels) or strong (+2; lower panels) staining intensity. Original magnifications x100.

**Figure 2:**
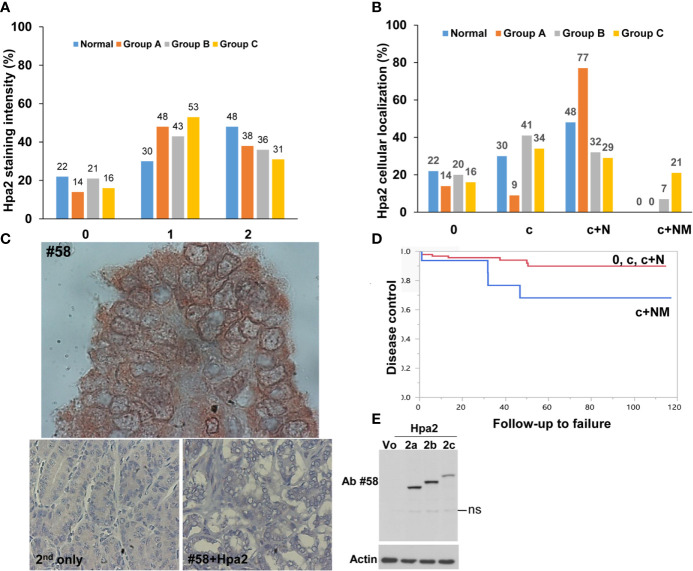
**(A)** Staining intensity of Hpa2 in the study groups. Hpa2 staining was scored according to its staining intensity (0- negative, 1- weak, 2- strong) in normal, benign (group A), non-metastatic (group B), and metastatic (group C) PTC. Shown is a graphical presentation of the percent of patients in each group according to the staining intensity of Hpa2. **(B)** Cellular localization of Hpa2. Hpa2 staining was scored according to its cellular localization (c- cytoplasm, c+N- cytoplasm and nucleus, c+NM- cytoplasm and nuclear membrane) in normal, benign (group A), non-metastatic (group B) and metastatic (group C) PTC. Shown is a graphical presentation of the percent of patients in each group according to the cellular localization of Hpa2. Note that NM Hpa2 is much more abundant in metastatic PTC (group C). **(C)** NM (nuclear membrane) localization of Hpa2. Metastatic PTC was subjected to immunostaining applying anti-Hpa2 antibody. Note, accumulation of Hpa2 immunoreactivity on the membrane of the cell nuclei. No immunostaining is observed once the primary antibody is omitted (lower left panel), or when purified Hpa2 protein (1 µg/ml) was added together with the primary antibody (#58+Hpa2; lower right panel). Original magnifications: upper panel x250, lower panels x100. **(D)** Plot of locoregional control stratified by cell distribution of Hpa2: c+NM (blue line) vs. other groups 0, c, c+N (red line), p=0.039. **(E)** Hpa2 splice variants. HEK 293 cells were transfected with control empty vector (Vo) or plasmids that carry the wild type, full-length Hpa2 (2c), or Hpa2 splice variants (2a, 2b) gene constructs. Cell lysate samples were subjected to immunoblotting applying anti-Hpa2 antibody #58 (upper panel) and anti-actin antibody (lower panel). ns- non specific.

To reveal the role of Hpa2 in PTC we subjected biopsies of benign, non-metastatic and metastatic PTC to immunostaining applying anti-Hpa2 antibody, and staining intensity was categorized as Hpa2-negative (0), weak (+1), or strong (+2) (**Figure 1**). Notably, we found that Hpa2 staining intensity does not significantly change in the transition from normal thyroid gland (**Figure 1**, left panels) to benign (**Figure 1**, goiter, second left), non-metastatic (second right) or metastatic (right panels) thyroid carcinoma. For each category, some biopsies were stained negative for Hpa2 (**Figure 1**, upper panels) and some were stained positive, exhibiting weak (**Figure 1**, middle panels) or strong (**Figure 1**, bottom panels) staining, but the overall staining pattern was similar among all study groups (**Table 1** and **Figure 2A**) and did not correlate with tumor size (p = 0.84), T-stage (p = 0.12) and the total number of positive lymph nodes (p = 0.64).

Follicular adenoma exhibited a higher percentage of weak staining (+1) for Hpa2 (71%), compared to Goiter (40.5%), PTC (43%), or metastatic papillary carcinoma (42%). Likewise, strong staining (+2) for Hpa2 was low in the follicular adenoma group (7%) as compared to goiter (47%), PTC (36%), or metastatic papillary carcinoma (30.6%), but these differences were statistically insignificant (p=0.16).

In addition to the expected localization of Hpa2 in the cell cytoplasm, it was also detected in the cell nucleus (**Figure 1**, middle panels) but, again, this localization appeared comparable among the study groups (**Figure 2B** and **Table 1**). Remarkably, we observed that in some biopsies, Hpa2 is accumulating on the membrane (envelop) of the nucleus and termed this cellular localization NM (nuclear membrane; **Figure 2C**, upper panel). Notably, no immunostaining is observed once the primary antibody is omitted (**Figure 2C**, lower left panel), or when purified Hpa2 protein (1 µg/ml) was added together with the primary antibody (**Figure 2C**, lower right panel). We found that NM localization of Hpa2 occurred primarily in metastatic PTC; none of the benign lesions (follicular adenoma and goiter) exhibited NM staining, compared to 7% of PTC and 21% of metastatic PTC, differences that were statistically highly significant (p<0.0001; **Figure 2B** and **Table 1**). Moreover, NM localization of Hpa2 was associated with a lower locoregional control rate (**Figure 2D**). Furthermore, NM Hpa2 correlated with an increased number of positive (metastatic) lymph nodes collected at surgery (F=3.5, p = 0.02) (**Table 2**); Borderline correlation was found between NM Hpa2 and tumor extra thyroid extension (ETE) (Pearson X2 = 6.4, p = 0.09).

**Table 2:** NM Hpa2 correlates with increased number of positive nodes in metastatic PTC (group C).

Cellular localization of Hpa2	Number of patients(n = 62)	Number of positive nodes (mean ± SEM)
0	10	10.4 ± 2.55
c	21	7.5 ± 1.8
c+N	18	6.8 ± 1.9
c+NM	13	**15.7 ± 2.2**

p = 0.02. Numbers in bold direct readers to the most important values.

In multivariate analysis for predicting the total number of positive lymph nodes in neck dissection, significant and independent parameters were age (p = 0.00031), NM Hpa2 (p = 0.00668), and tumor size (T; p = 0.01926). Thus, cellular localization of Hpa2 to the nuclear membrane appears as an important parameter that predicts patient failure in PTC.

## Discussion

Intensive research effort devoted in the last two decades to explore the significance of heparanase in cancer led to the recognition that heparanase is a valid drug target (6, 7, 9). As such, heparanase inhibitors are being evaluated clinically as anti-cancer therapeutics (10, 11). In contrast, little attention was given to its close homolog, Hpa2, possibly because it lacks HS-degrading activity typical of heparanase (13). Several lines of evidence suggest that unlike heparanase, Hpa2 functions to attenuate tumor growth. Hpa2 staining is evident in the normal epithelium of the bladder, breast, gastric and ovarian tissues. Notably, Hpa2 levels are reduced substantially in the resulting carcinomas (20, 21), a staining pattern typical of a tumor suppressor. Recently, Zhang et al. reported that Hpa2 gene methylation results in decreased Hpa2 expression (23). Importantly, hypermethylation of Hpa2 was associated with poor prognosis of colorectal cancer patients (23), thus further supporting the notion that Hpa2 functions to suppress tumorigenesis. Moreover, we have reported that in head & neck (H&N) cancer high levels of Hpa2 are associated with prolonged patients’ survival and decreased tumor cell dissemination to regional lymph nodes (13, 14). Also, overexpression of Hpa2 in H&N cancer cells resulted in a marked decrease in tumor growth, associating with a prominent reduction in tumor vascularity (blood and lymph vessels) likely due to reduced Id1 expression (15), a transcription factor highly implicated in VEGF-A and VEGF-C gene regulation (24). Tumors produced by cells overexpressing Hpa2 were not only smaller but also exhibited a higher degree of cell differentiation (15), further strengthening the significance of Hpa2 as a tumor suppressor (20, 21). In accordance with this notion, Hpa2 was found to attenuate the migration of primary (i.e., endothelial cells) and tumor-derived (i.e., 5637 bladder carcinoma) cells (25, 26), and to support cell adhesion. Surprisingly, exogenous addition of Hpa2 was noted to dissociate cell colonies (25), resulting in cell scattering effect that is considered to be pro-metastatic, raising the possibility that Hpa2 may function to promote tumorigenesis in certain tissues.

Our results indicate that in thyroid carcinoma, Hpa2 functions to promote lymph node metastasis once localized to the nuclear membrane. Hpa2 is expressed to relatively high levels in thyroid epithelium (**Figure 1**, left) but unlike carcinomas of the bladder, gastric, breast and ovarian tissues (20, 21), its expression was not altered in thyroid carcinoma (**Figure 2A**). Unlike the study of Matos et al (27), we did not find colloidal staining of Hpa2, possibly due to a different anti-Hpa2 antibody utilized. It may well be that the commercial anti-Hpa2 antibody utilized by Matos et al detects Hpa2 variants or modifications that are not detected by our antibody. It is also possible that the secreted Hpa2, accumulated in colloids, assumes a different conformation so that some epitopes are more or less exposed. These aspects, and the characterization of novel Hpa2 variants and modifications, are the subject of a separate study.

Notably, we observed that Hpa2 is accumulating on the nuclear membrane (NM; **Figure 2B**) of thyroid carcinoma cells, a unique cellular localization that has not been described for Hpa2 before. Remarkably, NM localization of Hpa2 prevailed in metastatic thyroid carcinoma (**Figure 2B**) and was associated with an increased number of infected (metastatic) lymph nodes (**Table 2**). Moreover, multivariate logistic regression analyses revealed that NM Hpa2 is an important parameter that predicts, at high statistical significance, the outcome of PTC patients. Thus, unlike previous results with H&N cancer patients where high levels of Hpa2 in the tumor lesions were associated with good prognosis (13), in thyroid carcinoma, Hpa2 appears to promote the disease when localized to the NM. In this regard, our results and the publication of Matos et al (27) come to a similar conclusion, linking Hpa2 to a more severe PTC disease.

It should be noted that the anti-Hpa2 antibody being employed (Ab #58) recognizes not only the wild type, full-length, Hpa2 protein (Hpa2c) but also Hpa2 splice variants Hpa2a and Hpa2b (22) (**Figure 2E**). Notably, Hpa2a and Hpa2b are not secreted (13) and their biological significance is unclear. It is therefore possible that one of these splice variants, reported to exist in thyroid carcinoma (27), or other spliced variants of Hpa2 described by others (28), is the one being localized to the NM. This possibility awaits the development of splice variant-specific antibodies. The nuclear lamina was described as a structure underlying the nuclear membrane that coordinates essential processes including DNA repair, genome organization, and epigenetic and transcriptional regulation. Loss of protein regulation (proteostasis) resulted in accumulation of protein aggregates within the lamina, affecting its integrity (29). Given the abundance of HS in the cell nucleus (30) and the high affinity of Hpa2 to HS (13), NM localization may involve the interaction of Hpa2 with HS on the nuclear membrane. Interestingly, the expression of heparanase was found to be substantially increased in PTC vs benign lesions, associating with increased metastasis (31). This suggests that in PTC, heparanase and Hpa2 may co-operate in driving tumor metastasis. This may involve physical interaction between the two proteins (13), interactions with HS in the cell nucleus and/or nuclear membrane, or independent function of heparanase and Hpa2. The nuclear membrane is also an integral part of the endoplasmic reticulum (ER) (32). Preliminary results indicate the Hpa2 promotes ER stress response in pancreatic carcinoma cells (21). NM Hpa2 may therefore result from such stress conditions that often occur in tumors due to the high proliferative rate and metabolic demands of cancer cells.

The results presented in this work provide new insight into Hpa2 cellular localization and its seemingly pro-tumorigenic properties in PTC. This opens new directions in Hpa2 research and mode of action, which by far is still lacking. Given the significance of the nuclear lamina in pathological conditions such as neurodegenerative diseases and aging (29), NM Hpa2 may turn important in pathologies and genetic disorders other than urofacial syndrome (33), but more work is required to study these aspects in detail.

## Data Availability Statement

The original contributions presented in the study are included in the article/supplementary material. Further inquiries can be directed to the corresponding authors.

## Ethics Statement

The studies involving human participants were reviewed and approved by Institutional Review Board, Carmel Medical Center. The patients/participants provided their written informed consent to participate in this study.

## Author Contributions

Conception and design: IV and NI. Development of methodology: MGC and IM. Acquisition of data: IM and IN. Analysis and interpretation of data: IM, ID, and NI. Writing, review, and/or revision of the manuscript: IN, NI, IV, and ID. Study supervision: IV. All authors contributed to the article and approved the submitted version.

## Funding

These studies were generously supported by research grants awarded to IV by the Israel Science Foundation (grant 1021/19); the Israel Cancer Research Fund (ICRF); and the Ministry of Science & Technology of the State of Israel and the German Cancer Research Center (DKFZ). IV is a Research Professor of the ICRF.

## Conflict of Interest

The authors declare that the research was conducted in the absence of any commercial or financial relationships that could be construed as a potential conflict of interest.

## References

[1] Khanna M Parish CR. Heparanase: Historical Aspects and Future Perspectives. Adv Exp Med Biol (2020) 1221:71–96. 10.1007/978-3-030-34521-1_3 32274707

[2] Mayfosh AJ Baschuk N Hulett MD. Leukocyte Heparanase: A Double-Edged Sword in Tumor Progression. Front Oncol (2019) 9:331. 10.3389/fonc.2019.00331 31110966 PMC6501466

[3] Blich M Golan A Arvatz G Sebbag A Shafat I Sabo E. Macrophage activation by heparanase is mediated by TLR-2 and TLR-4 and associates with plaque progression. Arterioscler Thromb Vasc Biol (2013) 33:e56–65. 10.1161/ATVBAHA.112.254961 PMC3548034 23162016

[4] Sanderson RD Bandari SK Vlodavsky I. Proteases and glycosidases on the surface of exosomes: Newly discovered mechanisms for extracellular remodeling. Matrix Biol (2019) 75-76:160–9. 10.1016/j.matbio.2017.10.007 PMC5920797 29106944

[5] Bhattacharya U Gutter-Kapon L Kan T Boyango I Barash U Yang SM. Heparanase and Chemotherapy Synergize to Drive Macrophage Activation and Enhance Tumor Growth. Cancer Res (2020) 80:57–68. 10.1158/0008-5472.CAN-19-1676 31690669 PMC6942624

[6] Jayatilleke KM Hulett MD. Heparanase and the hallmarks of cancer. J Transl Med (2020) 18:453. 10.1186/s12967-020-02624-1 33256730 PMC7706218

[7] Rivara S Milazzo FM Giannini G. Heparanase: a rainbow pharmacological target associated to multiple pathologies including rare diseases. Future Med Chem (2016) 8:647–80. 10.4155/fmc-2016-0012 27057774

[8] Sanderson RD Elkin M Rapraeger AC Ilan N Vlodavsky I. Heparanase regulation of cancer, autophagy and inflammation: new mechanisms and targets for therapy. FEBS J (2017) 284:42–55. 10.1111/febs.13932 27758044 PMC5226874

[9] Vlodavsky I Singh P Boyango I Gutter-Kapon L Elkin M Sanderson RD. Heparanase: From basic research to therapeutic applications in cancer and inflammation. Drug Resist Upd (2016) 29:54–75. 10.1016/j.drup.2016.10.001 PMC5447241 27912844

[10] Dredge K Brennan TV Hammond E Lickliter JD Lin L Bampton D. A Phase I study of the novel immunomodulatory agent PG545 (pixatimod) in subjects with advanced solid tumours. Br J Cancer (2018) 118:1035–41. 10.1038/s41416-018-0006-0 PMC5931096 29531325

[11] Galli M Chatterjee M Grasso M Specchia G Magen H Einsele H. Phase I study of the heparanase inhibitor roneparstat: an innovative approach for ultiple myeloma therapy. Haematologica (2018) 103:e469–e72. 10.3324/haematol.2017.182865 PMC6165822 29700168

[12] Doweck I Kaplan-Cohen V Naroditsky I Sabo E Ilan N Vlodavsky I. Heparanase localization and expression by head and neck cancer: correlation with tumor progression and patient survival. Neoplasia (2006) 8:1055–61. 10.1593/neo.06577 PMC1783722 17217623

[13] Levy-Adam F Feld S Cohen-Kaplan V Shteingauz A Gross M Arvatz G. Heparanase 2 interacts with heparan sulfate with high affinity and inhibits heparanase activity. J Biol Chem (2010) 285:28010–9. 10.1074/jbc.M110.116384 PMC2934666 20576607

[14] Doweck I Feibish N. Opposing Effects of Heparanase and Heparanase-2 in Head & Neck Cancer. Adv Exp Med Biol (2020) 1221:847–56. 10.1007/978-3-030-34521-1_37 32274741

[15] Gross-Cohen M Feld S Doweck I Neufeld G Hasson P Arvatz G. Heparanase 2 attenuates head and neck tumor vascularity and growth. Cancer Res (2016) 76:2791–801. 10.1158/0008-5472.CAN-15-1975 PMC4873389 27013193

[16] Tufano RP Noureldine SI Angelos P. Incidental thyroid nodules and thyroid cancer: considerations before determining management. JAMA Otolaryngol Head Neck Surg (2015) 141:566–72. 10.1001/jamaoto.2015.0647 25928353

[17] Robinson TJ Thomas S Dinan MA Roman S Sosa JA Hyslop T. How Many Lymph Nodes Are Enough? Assessing the Adequacy of Lymph Node Yield for Papillary Thyroid Cancer. J Clin Oncol (2016) 34:3434–9. 10.1200/JCO.2016.67.6437 PMC6366339 27528716

[18] Haugen BR. 2015 American Thyroid Association Management Guidelines for Adult Patients with Thyroid Nodules and Differentiated Thyroid Cancer: What is new and what has changed? Cancer (2017) 123:372–81. 10.1002/cncr.30360 27741354

[19] Amin SN Shinn JR Naguib MM Netterville JL Rohde SL. Risk Factors and Outcomes of Postoperative Recurrent Well-Differentiated Thyroid Cancer: A Single Institution’s 15-Year Experience. Otolaryngol Head Neck Surg (2020) 162:469–75. 10.1177/0194599820904923 32069184

[20] Ilan N Bhattacharya U Barash U Boyango I Yanku Y Gross-Cohen M. Heparanase-The message comes in different flavors. Adv Exp Med Biol (2020) 1221:253–83. 10.1007/978-3-030-34521-1_9 32274713

[21] Vlodavsky I Gross-Cohen M Weissmann M Ilan N Sanderson RD. Opposing Functions of Heparanase-1 and Heparanase-2 in Cancer Progression. Trends Biochem Sci (2018) 43:18–31. 10.1016/j.tibs.2017.10.007 29162390 PMC5741533

[22] McKenzie E Tyson K Stamps A Smith P Turner P Barry R. Cloning and expression profiling of Hpa2, a novel mammalian heparanase family member. Biochem Biophys Res Commun (2000) 276:1170–7. 10.1006/bbrc.2000.3586 11027606

[23] Zhang H Xu C Shi C Zhang J Qian T Wang Z. Hypermethylation of heparanase 2 promotes colorectal cancer proliferation and is associated with poor prognosis. J Transl Med (2021) 19:98. 10.1186/s12967-021-02770-0 33663522 PMC7934273

[24] Fong S Debs RJ Desprez PY. Id genes and proteins as promising targets in cancer therapy. Trends Mol Med (2004) 10:387–92. 10.1016/j.molmed.2004.06.008 15310459

[25] Gross-Cohen M Feld S Arvatz G Ilan N Vlodavsky I. Elucidating the consequences of heparan sulfate binding by heparanase 2. Front Oncol (2020) 10:627463. 10.3389/fonc.2020.627463 33585253 PMC7879983

[26] Gross-Cohen M Feld S Naroditsky I Nativ O Ilan N Vlodavsky I. Heparanase 2 expression inversely correlates with bladder carcinoma grade and stage. Oncotarget (2016) 7:22556–65. 10.18632/oncotarget.8003 PMC5008381 26968815

[27] Matos LL Suarez ER Theodoro TR Trufelli DC Melo CM Garcia LF. The Profile of Heparanase Expression Distinguishes Differentiated Thyroid Carcinoma from Benign Neoplasms. PloS One (2015) 10:e0141139. 10.1371/journal.pone.0141139 26488476 PMC4619411

[28] Vreys V David G. Mammalian heparanase: what is the message? J Cell Mol Med (2007) 11:427–52. 10.1111/j.1582-4934.2007.00039.x PMC3922351 17635638

[29] Almendariz-Palacios C Gillespie ZE Janzen M Martinez V Bridger JM Harkness TAA. The Nuclear Lamina: Protein Accumulation and Disease. Biomedicines (2020) 8(7):188. 10.3390/biomedicines8070188 PMC7400325 32630170

[30] Kovalszky I Hjerpe A Dobra K. Nuclear translocation of heparan sulfate proteoglycans and their functional significance. Biochim Biophys Acta (2014) 1840:2491–7. 10.1016/j.bbagen.2014.04.015 24780644

[31] Xu X Quiros RM Maxhimer JB Jiang P Marcinek R Ain KB. Inverse correlation between heparan sulfate composition and heparanase-1 gene expression in thyroid papillary carcinomas: a potential role in tumor metastasis. Clin Cancer Res (2003) 9:5968–79. 14676122

[32] Dultz E Ellenberg J. Nuclear envelope. Curr Biol (2007) 17:R154–6. 10.1016/j.cub.2006.12.035 17339009

[33] Roberts NA Woolf AS. Heparanase 2 and Urofacial Syndrome, a Genetic Neuropathy. Adv Exp Med Biol (2020) 1221:807–19. 10.1007/978-3-030-34521-1_35 32274739

